# The impact of bone channel entrance selection within the footprint region of the femoral anterior cruciate ligament on the achievement of near isometric ACL reconstruction in rabbits

**DOI:** 10.3389/fsurg.2025.1571180

**Published:** 2025-07-10

**Authors:** Jiating Zhang, Chengfeng Xu, Jiangtao Wang, Jia Zhang, Gang Zhao, Liu Liu, Chunbao Li, Yujie Liu

**Affiliations:** ^1^ Emergency Surgery Department, The Third Medical Center of Chinese PLA General Hospital, Beijing, China; ^2^ School of Graduate Studies, Chinese PLA Medical School, Beijing, China; ^3^ Senior Department of Orthopedics, The Forth Medical Center of Chinese PLA General Hospital, Beijing, China; ^4^ Physical and Chemical Laboratory, Chaoyang District Center for Disease Control and Prevention, Beijing, China; ^5^ Department of Orthopedics, Hainan Hospital of PLA General Hospital, Hai Nan, China

**Keywords:** ACL isometric reconstruction, femoral tunnel entrance, graft strain, tunnel position, isometric

## Abstract

**Introduction:**

Construction of an animal model of rabbit anterior cruciate ligament (ACL) near isometric reconstruction is a basic condition to study the patterns of graft stress and tendon to bone healing. The impact of alterations in the bone tunnel entrance within the femoral footprint region on graft tension remains uncertain. The objective of this study was to determine the femoral tunnel entrance that provides the closest approximation to near-isometric reconstruction within the femoral ACL footprint.

**Materials and methods:**

Eighteen Cadaveric rabbit knees were used in this experiment. The semitendinosus autografts were employed for ACL reconstruction. Six knees were reconstructed using the middle position of the femoral footprint area as the entrance (Mi-tunnel), six knees used a position 2 mm anterior to the middle (An-tunnel), and six knees used a position 2 mm posterior to the middle (Po-tunnel). All grafts were pretensioned with 10 N at 150° and 90° flexion and firmly fixed at the tibial end. The change in graft strain was measured under maximum flexion and extension in the rabbit knee joints.

**Results:**

Under a maximum flexion of 150° and 10 N pretension, the graft strain decreased significantly during knee extension in the An-tunnel and Mi-tunnel groups and there was no significant decrease in the Po-tunnel group. There were significant differences in graft strain between the Po-tunnel and An-tunnel during knee extension at 135°–35° (*P* < 0.05). Under 90° flexion and 10 N pretension, the Po-tunnel group showed a minimal change in graft strain compared to the An-tunnel and Mi-tunnel groups with knee extension and flexion (135°–35°) except at the initial pretension Angle. There was a statistically significant difference in graft tension when the Po-tunnel compared to the An-tunnel (*P* < 0.05).

**Conclusions:**

The Po-tunnel within the femoral footprint region may be the best choice for ACL near isometric reconstruction in rabbits.

## Introduction

Anterior cruciate ligament (ACL) injury is a common sports-related issue, with over 200,000 cases reported annually in the United States. The associated costs, both direct and indirect, exceed $7 billion each year, and ACL ruptures account for approximately 50% of all knee ligament injuries (1). ACL reconstruction is a widely accepted treatment for restoring motor function after ligament rupture, particularly in young patients with a high demand for physical activity (2, 3). Despite its recognized therapeutic efficacy (1, 4, 5), the incidence of osteoarthritis after reconstruction remains high (5, 6). Studies have identified several factors contributing to joint instability and poor tendon-bone healing after ACL reconstruction, such as the fit between the graft and tunnel (7), graft fixation method (8), graft position in the tunnel, and mechanical stress (9, 10). Tendon-bone healing is influenced by surgical techniques, graft selection, and postoperative rehabilitation (11). Given the growing maturity of surgical methods with similar grafts, more attention has been directed towards the impact of mechanical stress during postoperative rehabilitation on tendon-bone healing (12–14). Therefore, it is crucial to explore the mechanisms of osteoarthritis following ACL injury and the factors contributing to poor tendon-bone healing through animal models to identify new interventions.

Preclinical studies using small laboratory animals like mice, rats, and rabbits are essential for evaluating factors affecting graft bone growth and osteoarthritis after ACL reconstruction (15). Previous research has indicated that different femoral tunnel entrances significantly affect ACL graft strain and strain in rats (16). Mechanical tests can help identify the optimal tunnel for isometric reconstruction. Rabbits are well-suited for ACL reconstruction experiments (15). However, there are anatomical and functional differences between rat and rabbit knee joints, including the number of ACL bundles, tibial plateau angle, range of motion, ACL force, and gai (15). As a result, the variation in ACL with knee flexion and extension is different between rats and rabbits. While there are some studies on ACL isometric reconstruction in rabbit models (17), detailed investigations on the anatomical characteristics of the femoral insertion of the rabbit ACL and the effects of various tunnels in the footprint area on graft strain during knee flexion and extension are limited. Thus, it is essential to study the impact of the femoral tunnel entrance on graft strain and the patterns of graft strain variation during flexion and extension in the rabbit ACL reconstruction model.

Furthermore, the topic of anatomic ACL reconstruction methods, specifically single-bundle ACLR and double-bundle ACLR, remains highly debated (18, 19). However, it is crucial to consider that rat ACL has two bundles, while rabbit ACL has one bundle (15). When using rabbits for double-bundle ACLR experiments, it becomes particularly important to understand the strain variation patterns of the graft during reconstruction using various tunnels in the footprint area as the femoral tunnel entrance.

This study provides a comprehensive rabbit model of ACL reconstruction to investigate several key aspects: (1) the anatomical characteristics of the femoral insertion in rabbits; (2) the successful preparation of the femoral tunnel in the central and anterior locations of the original ACL footprint area; (3) the significant differences in graft strain with various tunnels in the footprint area as the femoral tunnel entrance during ACLR; and (4) the variation patterns of graft strain during full range flexion and extension in ACLR with different tunnel entrances in the footprint area.

## Methods

All animal experiments were conducted in accordance with the guidelines and approved by the Animal Experiment Ethics Committee of the Hospital. We used the R package “pwr” to calculate the sample size. In the present study, we compared the force between the “An-tunnel”, “Mi-tunnel”, and “Po-tunnel”, when the knee angle was in 75 degrees, the pretension of the knee joints was set at 90 flexion. The result showed the means of the 3 groups were 7.37, 8.71, and 9.91, and the SDs of the 3 groups were 0.63, 0.48, and 0.24. We used “sig.level” was 0.05, and power was 0.9. In the R package “pwr”, the value of ψ is provided by the package. At last, the results showed that *n* = 2. Considering the possible bias, we expanded the sample size to 6. For this study, eighteen male New Zealand white rabbits (2.5–3.5 kg, with bone maturity) were used, and their intact knee joints were obtained after completion of other experiments. The knee joints were randomly divided into three groups: Group 1 underwent ACL reconstruction with the femoral tunnel entrance positioned 2 mm in front of the middle position of the footprint area of the femur (An-tunnel) (*n* = 6), Group 2 with the entrance at the middle position of the footprint area (Mi-tunnel) (*n* = 6), and Group 3 with the entrance 2 mm behind the middle position of the footprint area (Po-tunnel) (*n* = 6). Subsequently, all grafts were pretensioned to 10 N with the knee joints at 150 and 90 flexion and securely fixed at the tibial end. The change in graft strain was then measured during the maximum range of flexion and extension in the rabbit knee joints.

### Determination of maximum range of flexion and extension in rabbit knee joints

**Figure 1:**
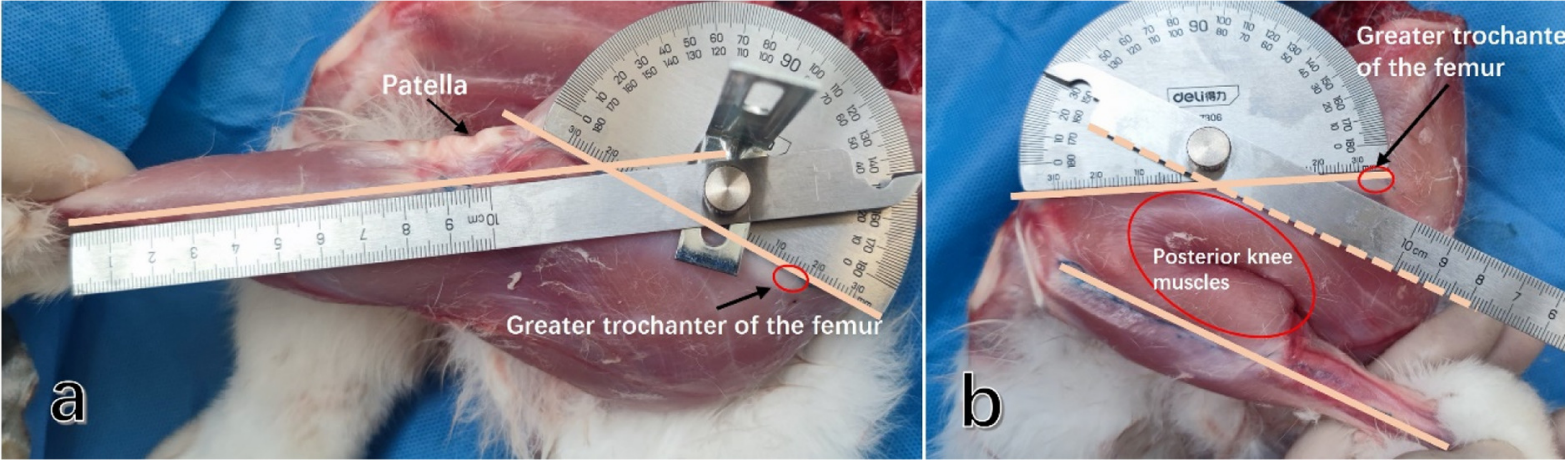
The maximum range of flexion and extension in the rabbit knee joint was determined by measuring the angle between the long axis of the femur and the long axis of the tibia. **(a)** The maximum extension angle of the rabbit knee joint was measured. **(b)** The maximum flexion angle of the rabbit knee joint was measured. (It was observed that the maximum knee flexion angle is influenced by the muscles located behind the lower extremities).

To determine the actual maximum range of flexion and extension in rabbit knee joints, we collected the intact lower limb below the pelvis of the rabbit, ensuring that all muscle tissues around the thigh and lower leg were retained. We marked the femoral axis on the body surface at the femoral greater trochanter and the femoral insertion of the lateral collateral ligament. Simultaneously, we marked the tibial axis starting from the tibial insertion of the lateral collateral ligament along the long axis of the femoral shaft to maximize knee joint flexion and extension. By measuring the angle between these two axes, we obtained the maximum range of flexion and extension (Figure 1).

The average value measured from 18 male New Zealand white rabbits (with bone maturity, weighing 2.5–3.5 kg) was taken as the final range.

### Anatomical characteristics of ACL in rabbits and selection of femoral tunnel entrance

**Figure 2:**
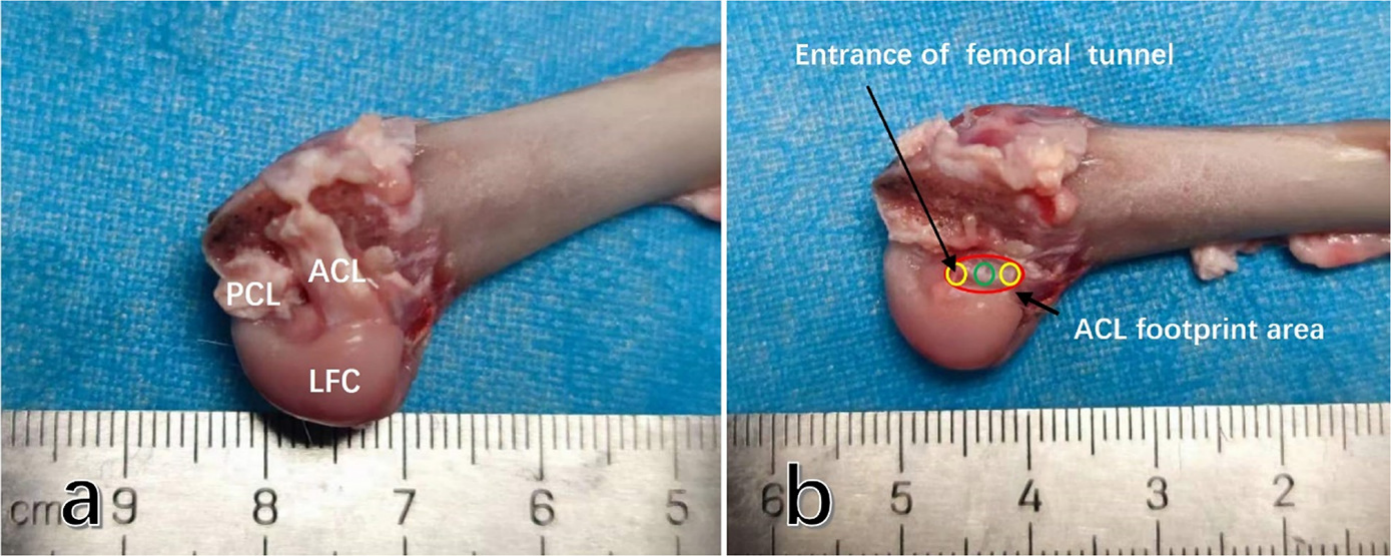
The anatomic characteristics of the anterior cruciate ligament (ACL) femoral insertion in rabbit knee joints are as follows: **(a)** after the removal of the condylus medialis femoris, it can be observed that the rabbit ACL is flat and terminates at the lateral femoral condyle of the femur. **(b)** The footprint area of the rabbit ACL is an oblate oval shape, and the anteroposterior diameter of the insertion is approximately half of that of the cartilage margin in the medial femoral condyle. Abbreviations used: ACL, anterior cruciate ligament; PCL, posterior cruciate ligament; LFC, lateral femoral condyle.

We selected ten male New Zealand white rabbits (with bone maturity, weighing 2.5–3.5 kg) to obtain the knee joint specimens. The femur was retained while removing the femoral medial condyle. We observed the anatomical characteristics of the anterior cruciate ligament (ACL) and the femoral insertion. At the midpoint, we measured the anteroposterior diameter of the ACL and the diameter of the femoral footprint area. Subsequently, we marked the different femoral tunnel entrances using a 2 mm Kirschner wire (designated as Mi-tunnel, An-tunnel, Po-tunnel) (Figure 2). The final value used for analysis was the mean measured value from these ten knee joints.

### Rabbit anterior cruciate ligament reconstruction with semitendinosus tendon autograft

All 18 rabbit knee joints were completely dissected from the deceased rabbits, preserving the intact femur and its distal structures. The fur was removed, leaving the muscle tissue and knee capsule intact. The gracilis muscle was cut longitudinally from the posterior medial knee joint to expose the semitendinosus and its medial attachment point on the knee joint. The semitendinosus was carefully detached from the attachment point, and its tendon end was secured with a 3-0 ETHIBOND locking Krackow stitch (Ethicon, Inc.) to achieve satisfactory traction fixation. The stitch was gradually moved to expose the distal end of the semitendinosus up to the tendon-muscle transitional part, where the semitendinosus was transected. The tendon end was then secured again with a 3-0 ETHIBOND locking Krackow stitch (Ethicon, Inc.) to ensure satisfactory traction fixation.

**Figure 3:**
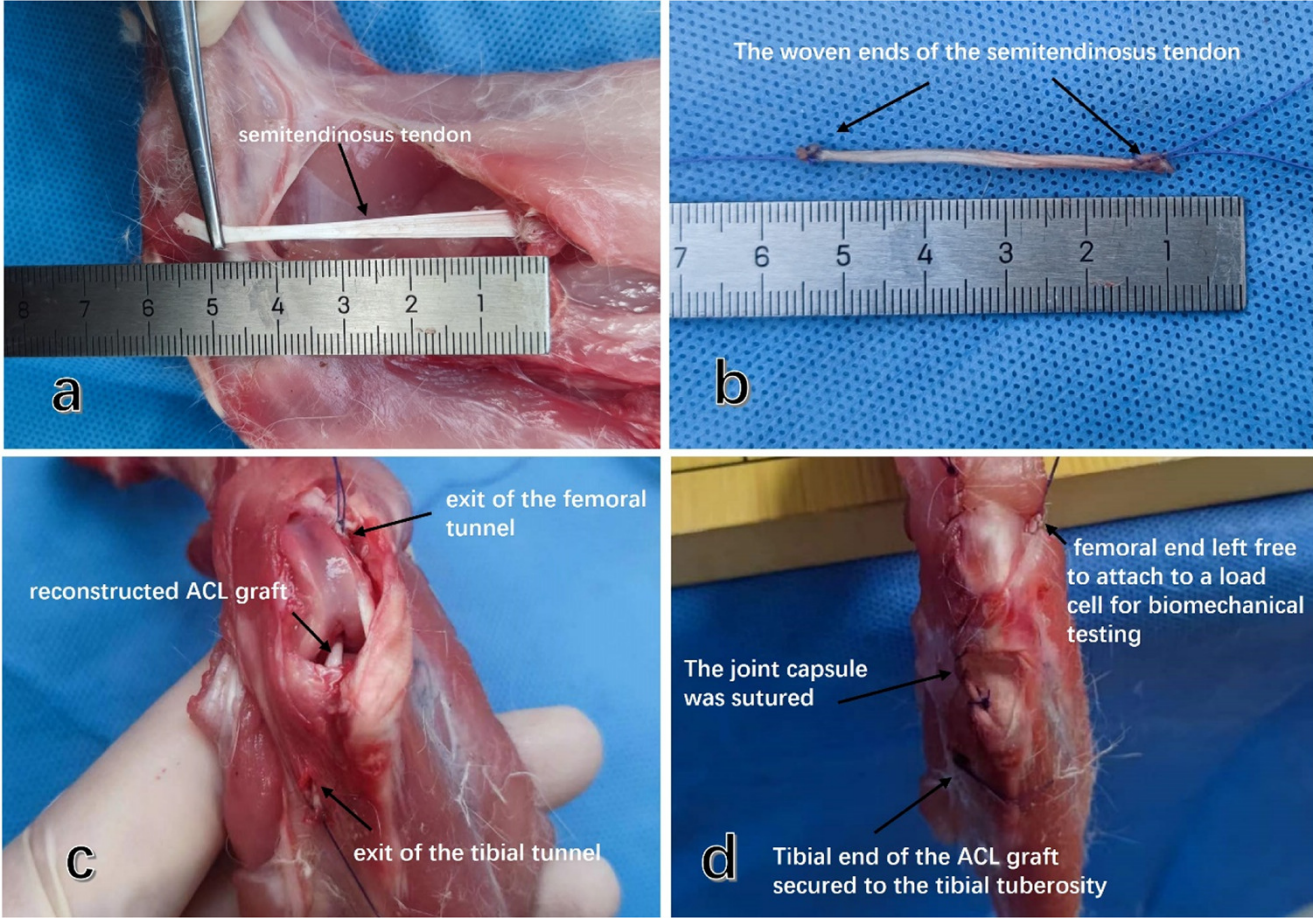
Rabbit ACL reconstruction using autosemitendinosus tendon and preparation of graft strain measurement. **(a)** The posterior medial free semitendinosus tendon of the knee joint was severed at the muscular tendon transition. **(b)** Both ends of the isolated semitendinosus graft were braided with 3-0 ETHIBOND locking Krackow stitches to facilitate subsequent fixation and graft pulling. **(c)** Grafts were pulled out from the joint by traction lines from the femoral tunnel entrance and the tibial tunnel entrance to reconstruct the ACL. **(d)** Holes were drilled in the tibial tubercle at the tibial outlet, and the tibial end of the graft was firmly fixed.

The semitendinosus autograft was observed and measured, and the diameter of both ends and middle parts was approximately 2.0 mm, demonstrating good consistency in graft diameter (Figures 3a,b).

The femur end of the graft was left free, allowing it to penetrate the anterolateral muscle of the femur and be pulled and fixed along the long axis of the bone tunnel to the mechanical tester. The articular capsule and the anterior medial muscle incision of the knee joint were sutured layer by layer.

After preparing the graft, it was covered with wet gauze for further operation. Throughout the procedure, all samples were kept moist with wet gauze to minimize the influence of muscle atrophy through water loss on knee joint activity.

**Figure 4:**
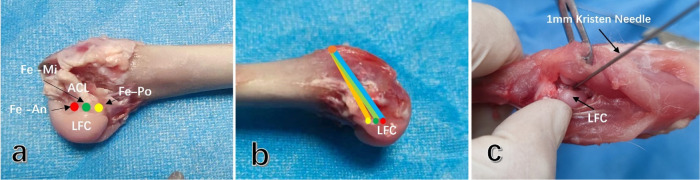
Selection and preparation of different femoral tunnel entrances in rabbit ACL. **(a)** The entrances of different femoral tunnels were located in the anterior (Fe-An), middle (Fe-Mi), and posterior (Fe-Po) footprint area of the ACL. **(b)** The lateral view of the femoral tunnel prepared with different entrances. **(c)** The entrance of the femoral tunnel behind the knee joint was observed directly, and a 1 mm Kirschner wire was used to accurately locate the entrance by drilling through the cortex. Subsequently, the femoral tunnel was prepared with a 2.0 mm Kirschner wire. ACL, anterior cruciate ligament; PCL, posterior cruciate ligament; LFC, lateral femoral condyle.

A medial parapatellar approach was employed to incise the joint capsule and widen the incision until the patella could be successfully pushed outward for dislocation. The ACL in the knee joint was then identified, and the sharp blade was carefully used to detach the ACL from its tibial insertion, ensuring that other important structures such as the meniscus, medial and lateral collateral ligaments, and posterior cruciate ligament were not damaged. An anterior drawer test was performed. Due to the narrow space of the trochlear in the rabbit's femoral knee, to ensure accurate positioning of the bone tunnel entrance, posterior dislocation of the femoral end of the knee was performed after the ACL was severed, allowing for blunt separation of the gastrocnemius space and incision of the posterior joint capsule. This facilitated easy visualization of the lateral intercondylar fossa of the femur. The femoral ACL attachment point was located, and the ACL remnant was carefully and completely removed with a sharp blade. In the different groups, 1 mm Kirschner wires were used to pre-drill holes at the middle, 2 mm in front, and 2 mm in the back of the footprint area of the ACL (Figures 4a,b), penetrating the cortex to ensure accurate positioning and prevent the bone drill from slipping during further drilling of the bone tunnel (Figure 4c).

Subsequently, 2.0 mm Kirschner wires were used to drill holes anterolaterally from the marks and exit behind the superior lateral margin of the trochlea of the femur. The exit point of the femoral tunnel is positioned 2 mm anterior to the cartilage edge of the femoral condyle, facilitating the insertion of the Kirschner wire without causing cortical bone fracture. This location also allows for the traction line to be aligned parallel to the tunnel, thereby minimizing frictional interference between the transplanted tendon and the tunnel wall. Femoral tunnels with different entrances were ultimately prepared. After the femoral tunnel preparation, a 2.0 mm Kirschner wire was inserted into the central area of the tibial attachment point, and the distal hole was drilled. The Kirschner wire was then inserted through the distal insertion of the medial collateral ligament to prepare the tibial tunnel.

After preparing the bone tunnel, the distal end of the 3-0 ETHIBOND locking Krackow stitch was utilized as the traction end, and a bone bridge was created from within the joint towards the distal bone canal of the tibia, allowing the graft to pass through the bone canal. The bone bridge was made under the tibial tubercle using a 1 mm Kirschner needle. The traction wire was then threaded through the bone canal of the bone bridge and tied for fixation. This traction wire emerged from the bone tunnel of the distal tibia from an intra-articular direction, with the graft passing through the tibial tunnel. Similarly, a bone bridge was created under the tibial tubercle with a 1 mm Kirschner wire, and the traction line passed through the bone bridge and bone tunnel, securing it with a knot for fixation. The femoral graft also emerged within the joint. To facilitate further measurement of graft strain, the femoral end of the graft was not fixed (Figure 3c). The traction line passed through the femoral cortex and anterolateral muscles, and it was fixed to the element of the mechanical testing machine. To bring the data measurements closer to those of live animals, we carefully re-closed the anterior and posterior incisions of the joint capsule after reconstruction, preserving the anterior femur muscle group to ensure the integrity of the patellar support band (Figure 3d).

### Biomechanical test of ACL graft

**Figure 5:**
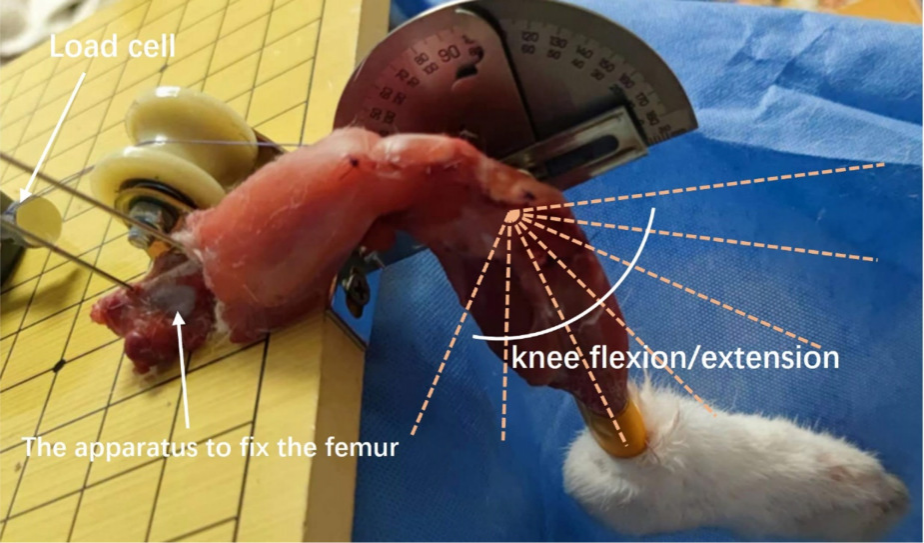
Measurement of graft strain under different knee flexion angles was conducted using the following method: the femur was secured on a fixed platform using two 1.5 mm kirschner wires penetrating through the double-layer cortex. The distal tibia was bound and fixed with the angle indicator. Different knee joint flexion and extension angles were achieved by flexion and extension of the tibia around the knee joint. The femoral end of the graft was fastened to the tension instrument with a traction line parallel to the long axis of the femoral tunnel.

This study utilized a fixed platform to investigate the change in graft strain during rabbit knee flexion and extension. Firstly, the femur was secured on the fixed platform using two 1.5 mm Kirschner wires penetrating through the double-layer cortex. The pull wire of the femoral end of the graft was fastened to the fixed port of the tensile testing machine, and the angle of the pull wire was adjusted to ensure that the pulling direction was parallel to the axis of the bone tunnel, reducing friction between the graft and the tunnel (Figure 5).

The graft was repeatedly pulled with an appropriate traction force until the force stabilized, and the tensile testing machine was fixed at the predetermined knee flexion angle, allowing the graft to obtain a pretension of 10 N. The graft strain remained stable at 10 N during the initial flexion angle and throughout the small range of flexion and extension.

To determine the flexion and extension angle of the knee joint, a pointer fixed parallel to the axis of the rabbit tibia and an angle gauge fixed to the test platform were used. After fixation at 90 with a pretension of 10 N, the knee joint was flexed and extended in 15 intervals, and the graft strain was measured and recorded. Similarly, after fixation at 150 with a pretension of 10 N, the knee joint was gradually extended in 15° intervals, and the graft strain was measured and recorded. Graft strain was tested under three different femoral tunnel entrances.

### Statistical analysis

Statistical significance was defined as *p*-value < 0.05 (two tails).

In order to analyze the change in graft strain after ACL reconstruction with different femoral tunnel entrances within a small range of ACL femoral footprints, the range of flexion and extension was examined. This study utilized the rank sum test of multiple independent samples with *post hoc* Bonferroni test to analyze the differences among the three groups. All statistical analyses were conducted using Rstudio software (R version 3.62), and the graphical representations were generated using the “ggplot2” package. Statistical significance was defined as a *p*-value < 0.05 (two-tailed).

## Results

### Maximum range of flexion and extension in rabbit knee joints

The results demonstrated that the rabbit knee joint had a maximum extension angle of 35° and a maximum flexion angle of 150°, unlike human knee. This difference might be attributed to the functional requirements of four-legged walking and the bouncing ability of hind limbs in rabbits. Notably, the development of lower limb muscles in rabbits had a significant impact on the range of flexion and extension. Therefore, to better simulate the knee joint in its living state, it was essential to retain all the lower limb muscle groups during the assessment of the maximum range of flexion and extension. Considering that the rabbit knee joint was flexed under the rest state, which was close to the maximum flexion position, we selected an initial angle of 90° for extension and a maximum flexion of 150° to measure the change in graft strain.

### Anatomical characteristics of rabbit ACL, selection of femoral tunnel entrance

The anatomical characteristics of the rabbit ACL were observed in 10 rabbit knee joints, revealing that the ACL had an oblate oval strip structure with a mean length of 9.1 mm and a mean anteroposterior diameter of 4.2 mm, approximately half of the anteroposterior diameter of the lateral femoral intercondylar fossa (Figure 2a). The mean anteroposterior diameter of the femoral footprint area was 4.4 mm (Figure 2a). Within the footprint area, a femoral tunnel with a diameter of 2 mm could be successfully prepared at the middle position, 2 mm in front of the middle position, and 2 mm behind the middle position (Figure 2b).

### Changes of ACL graft strain under pretension of 10 N with initial flexion of 150°

**Figure 6:**
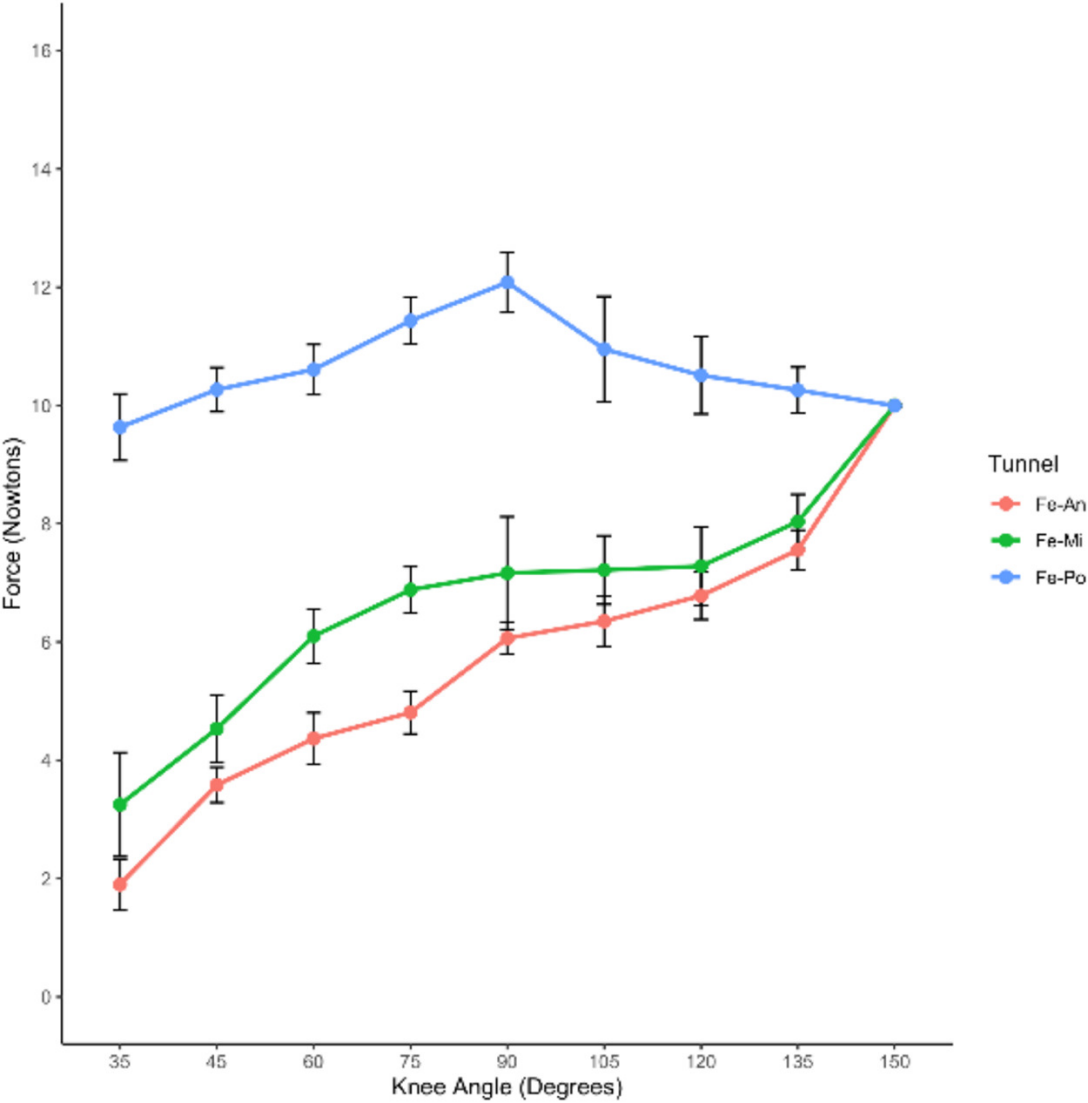
Changes in ACL graft strain were examined under a pretension of 10 N with an initial flexion of 150°. The results are presented in Table 1, showing the ACL graft strain in rabbit knee joints at various flexion and extension angles under the specified pretension conditions.

The changes in ACL graft strain were analyzed under a pretension of 10 N with an initial flexion of 150° (Table 1). The Po-tunnel group demonstrated relatively isometric graft behavior during an extension of 35°, and the strain change was not significant (Figure 6).

**Table 1:** ACL graft strain in rabbit knee joints at different flexion and extension angles under pretension of 10 N with initial flexion of 150°.

Knee angle (°)	Sample size	An-tunnel		Mi-tunnel		Po-tunnel		*P*	An-Mi tunnel	Mi-Po tunnel	An-Po tunnel
		Mean	Sd	Mean	Sd	Mean	Sd				
35	6	1.90	0.42	3.25	0.88	9.63	0.56	<0.05	0.22	0.13	<0.05
45	6	3.58	0.29	4.53	0.56	10.26	0.37	<0.05	0.25	0.12	<0.05
60	6	4.36	0.43	6.10	0.46	10.60	0.42	<0.05	0.15	0.15	<0.05
75	6	4.80	0.36	6.88	0.39	11.43	0.39	<0.05	0.15	0.15	<0.05
90	6	6.06	0.27	7.16	0.95	12.08	0.50	<0.05	0.58	0.07	<0.05
105	6	6.35	0.42	7.21	0.58	10.95	0.89	<0.05	0.31	0.10	<0.05
120	6	6.78	0.40	7.28	0.66	10.51	0.65	<0.05	1.00	<0.05	<0.05
135	6	7.55	0.33	8.03	0.45	10.26	0.38	<0.05	0.64	0.06	<0.05
150	6	10.00	0.02	10.00	0.01	9.99	0.01	0.77	—	—	—

The graft strain was highest at 90° flexion (12.08 ± 0.50 N) and lowest at maximum extension (9.63 ± 0.56 N). Under the pretension of 10 N with an initial flexion of 150°, both the Mi-tunnel and An-tunnel groups exhibited a significant downward trend in graft strain during extension, with the An-tunnel group showing a more substantial decrease compared to the Mi-tunnel group. As the knee joint was gradually extended to 35°, the graft strain in both the Mi-tunnel and An-tunnel groups reached its lowest values (1.90 ± 0.42 N in the An-tunnel group and 3.23 ± 0.88 N in the Mi-tunnel group), with no significant difference between these two groups.

Within the maximum range of extension, there was no significant difference in graft strain between the Mi-tunnel and An-tunnel groups. However, the Po-tunnel group exhibited a significant difference in graft strain from the beginning to an extension of 135° compared to the other two groups. These results may indicate that the Po-tunnel showed better isometric characteristics, suggesting that the fiber bundle behind the ACL might play the primary pulling role at maximum extension, despite the presence of one bundle in the rabbit ACL.

### Changes of ACL graft strain under pretension of 10 N with initial flexion of 90°

Under a pretension of 10 N with an initial flexion of 90°, different strain changes were observed in the ACL grafts of the three groups as the knee gradually extended to 35° (Table 2).

**Table 2:** ACL graft strain in rabbit knee joints at different flexion and extension angles under pretension of 10 N with initial flexion of 90°.

Knee angle (°)	Sample size	An-tunnel		Mi-tunnel		Po-tunnel		*P*	An-Mi tunnel	Mi-Po tunnel	An-Po tunnel
		Mean	Sd	Mean	Sd	Mean	Sd				
35	6	2.30	0.54	5.62	1.17	8.95	0.53	<0.05	0.16	0.16	<0.05
45	6	4.21	0.92	6.35	0.32	9.41	0.39	<0.05	0.16	0.16	<0.05
60	6	5.69	0.47	7.15	0.43	9.55	0.26	<0.05	0.20	0.13	<0.05
75	6	7.35	0.61	8.67	0.46	9.77	0.23	<0.05	0.28	0.11	<0.05
90	6	9.99	0.01	10.00	0.01	9.99	0.01	0.48	—	—	—
105	6	11.66	0.70	9.89	0.40	9.76	0.19	<0.05	<0.05	1.00	<0.05
120	6	11.71	0.94	10.30	0.72	9.10	0.22	<0.05	0.39	0.14	<0.05
135	6	12.09	0.63	11.06	0.49	8.06	0.51	<0.05	0.28	0.11	<0.05
150	6	12.91	2.85	11.37	0.77	7.36	0.86	<0.05	0.70	0.09	<0.05

**Figure 7:**
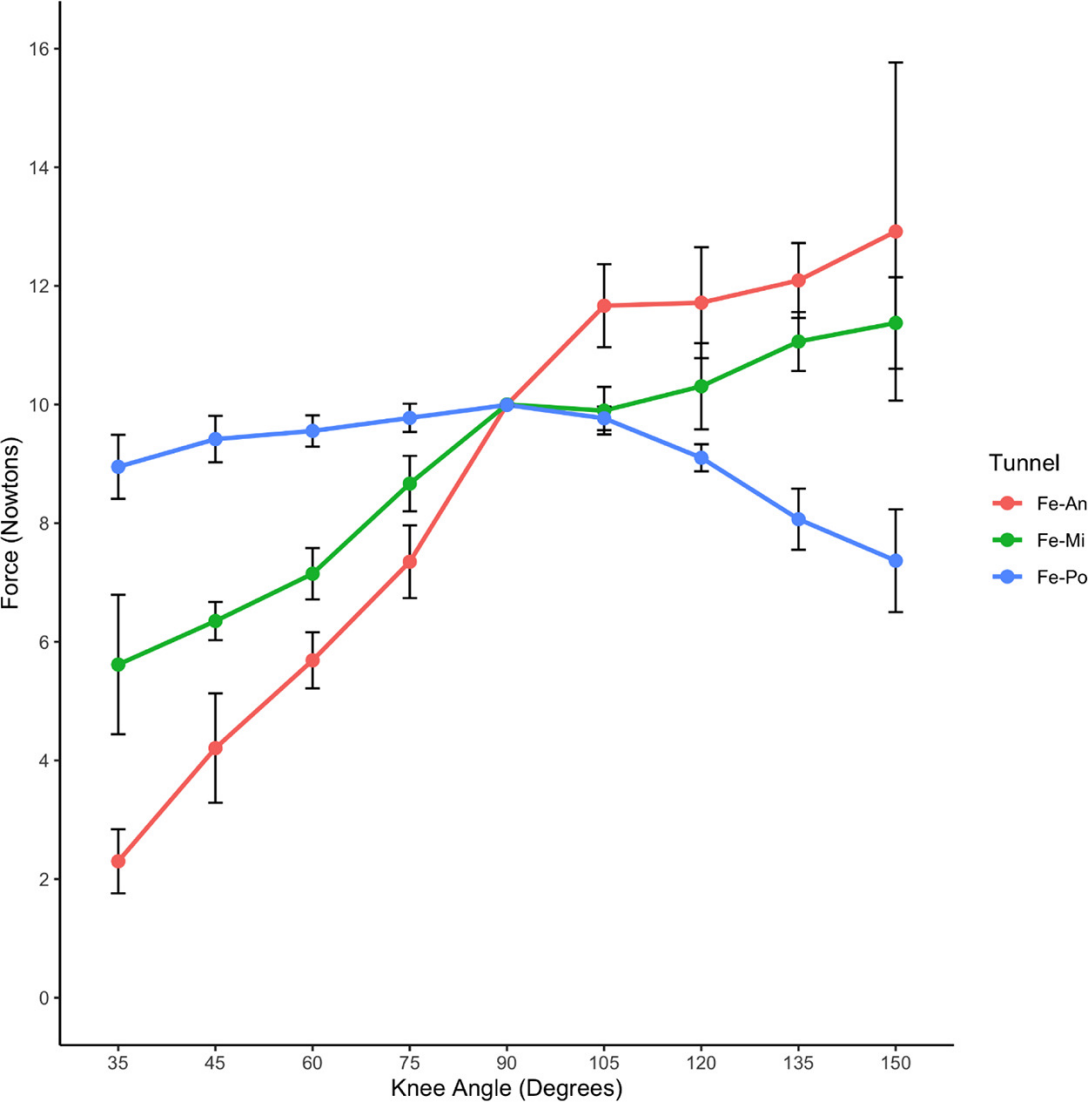
Changes of ACL graft strain under pretension of 10 N with initial flexion of 90°.

In the An-tunnel group, the graft strain showed a significant decrease with knee extension, reaching approximately 75% reduction (2.30 ± 0.54 N) compared to the baseline 10 N at an extension of 35°. This trend was consistent with the Mi-tunnel group, where the graft strain decreased by about 50% (5.62 ± 1.17 N) at an extension of 35°, but to a lesser extent than the An-tunnel group. In contrast, the Po-tunnel group exhibited no significant decrease in graft strain with gradual extension to 35°, with the graft strain measuring 8.95 ± 0.53 N at maximum extension. As the knee was gradually flexed to 150°, the An-tunnel group showed the most significant increase in graft strain, reaching 12.91 ± 0.85 N at the maximum flexion angle of 150°. The Mi-tunnel group exhibited a slight increase in graft strain (11.37 ± 0.77 N) without significant differences. Conversely, in the Po-tunnel group, the graft strain gradually decreased with increasing flexion angle, measuring 7.36 ± 0.86 N at the maximum flexion angle of 150° (Table 2). In the maximum range of flexion and extension, the Po-tunnel group demonstrated the smallest change in graft strain, indicating it was the closest to isometric reconstruction (Figure 7). These results also suggested that the anterior fiber bundle may play the primary pulling role at maximum flexion.

## Discussion

The construction of an animal model is a crucial step in studying the factors influencing tendon-bone healing after ACL reconstruction, and the New Zealand white rabbit is commonly chosen by researchers (20, 21). However, in many studies, the selection of the femoral tunnel entrance has not been adequately explained, and the establishment of tibial and femoral tunnels may be relatively poor. While previous reports indicated that rabbit ACL has only one bundle, our study found that the transverse section of rabbit ACL is elliptical, with a short yet broad length. Its anterior-posterior diameter accounts for approximately 50% of the anterior-posterior diameter of the femoral condyle (Figure 2a).

Moreover, in comparison to the human knee joint, the femoral condyle's footprint area is relatively larger in rabbits. Additionally, the narrow space of the femoral condyle in the rabbit knee joint poses challenges during the establishment of the femoral tunnel through the traditional anterior knee joint approach. Kirschner wires may be obstructed by the femoral condyle, tibial platform, and posterior cruciate ligament, leading to potential deviation in tunnel preparation. For small knee joints and short ACLs in rabbits, even slight selection bias in femoral tunnel entrance within a small range can have an amplified effect on the strain changes of ACL grafts. Hence, this study focused on the accurate selection of femoral tunnel entrances for ACL reconstruction based on the anatomical characteristics of rabbit knee joints. It was observed that even a slight deviation of 2 mm between different femoral tunnel entrances could result in distinct graft strain variations. The results of our study suggest that the Po-tunnel group showed a minimal change in graft strain compared to the An-tunnel and Mi-tunnel groups with knee extension and flexion (135°–35°). There was a statistically significant difference in graft tension when the Po-tunnel compared to the An-tunnel (*P* < 0.05)except at the initial pretension Angle. Although the difference of graft tension between the Po-tunnel and the Mi-tunnel was not statistically significant, it could be observed that the change range in the Po-tunnel group was the smallest through the graft stress curve. This suggests that the entrance of the bone tunnel should be as close as possible to the posterior cartilage margin of the femoral external condyle during tunnel preparation when we want to obtain an ACL near isometric reconstruction rabbit model. This underscores the importance for researchers to pay meticulous attention to the precise selection of the femoral tunnel entrance when studying tendon-bone healing. By doing so, the exploration of the effects of different factors on tendon-bone healing becomes more scientifically rigorous.

Regarding the construction of different femoral tunnel entrances, this study encountered challenges due to the narrow space of the rabbit knee femoral intercondylar fossa. The medial parapatellar approach made it difficult to accurately locate the femoral tunnel in the front of the joint and observe the femoral footprint area of ACL as clearly as in full endoscopic reconstructions of the human knee joint. To address this, we adopted the technique of backward dislocation of the femoral end after ACL resection in cadaveric specimens. By cutting the posterior joint capsule of the knee joint from the posterior muscle space, we were able to fully expose the femoral intercondylar fossa and accurately locate the femoral tunnel entrance under direct vision. Although drilling the femoral tunnel from the rear of the knee joint is challenging in live animals, the preparation of the femoral tunnel behind the knee joint holds promise for achieving accurate entrance and maximizing the potential for ACL isometric reconstruction, thus promoting tendon-bone healing. This method is worthy of further exploration and experimentation.

There is still some debate regarding the impact of mechanical force on tendon healing after ACL reconstruction. While some researchers argue that early restorative exercise and force application can promote the healing of the reconstructed graft and bone (22), others suggest that early immobilization may play a more significant role in tendon healing. To better understand the effect of mechanical stimulation of varying duration and force on tendon-bone healing, this study aimed to optimize experimental design and utilize more ideal animal models. Through experiments on isolated specimens, we found that the Po-tunnel approach allows for nearly isometric conditions during flexion and extension in rabbits. This implies that the femoral tunnel entrance should be positioned as close as possible to the cartilage margin in the medial lateral condyle to minimize graft strain interference during the construction of the ACL reconstruction rabbit model.

However, this study does have some limitations. When determining the range of flexion and extension in rabbit knee joints, direct measurement of passive knee joint motion using isolated lower limb specimens may not entirely replicate the actual range of knee joint motion observed in active animals during extreme activities. Additionally, as this study was conducted on cadaver knee specimens, certain knee joint structures were partially removed, and some joint capsule structure was also disturbed. In live New Zealand white rabbits, intact knee joint structures and maintained muscle tension could impact ACL strain. Despite our efforts to ensure that the pulling direction aligns with the femoral tunnel's axial direction to minimize frictional resistance, it is important to acknowledge that the femoral tunnel orientation may not consistently align with the axial direction of the graft during the flexion and extension phases of the knee joint. Furthermore, the three femoral tunnels in our experimental setup were not entirely parallel, which may have introduced variability in the experimental results due to differing frictional forces. This issue warrants further attention in subsequent studies to ensure more precise control over the experimental conditions. Furthermore, the use of the 3-0 ETHIBOND locking Krackow stitch to pull the graft and connect it to the mechanical testing instrument may not precisely replicate the mechanical properties of the transplanted tendon, potentially affecting graft strain measurements.

Moreover, ACL strain in the knee joint may vary under different states, such as weight-bearing, non-weight-bearing, rest, and motion states. Consequently, the ACL strain measured at different flexion states in this study may slightly differ from the actual strain changes in living animals. Another limitation is the variability in the position and area of the ACL footprint area in different rabbits. Given the small size of the knee joint and the relatively short length of the natural ACL, even minor differences in the location of the bone tunnel entrance can lead to distinct ACL strain variations. These are important factors that warrant attention and understanding.

Further studies, however, are necessary for the translation of the method *in vivo* on human knees. It can still be a point of reflection for surgeons in order to optimize the results of anterior cruciate ligament reconstruction. This study can only serve as a point of reflection rather than an absolute indication for the positioning of the femoral tunnel in human knee anterior ligament reconstruction. It is still mainly a reference for researchers in the construction of animal models of ACLR.

In conclusion, for ACL reconstruction in rabbits, the selection of different femoral tunnel entrances within the femoral footprint area can result in varying postoperative graft strain. This is a basis for animal experimentation to further understand the effect of graft stress on tendon bone healing after ACL reconstruction and to further guide postoperative rehabilitation. Choosing the entrance behind the middle point within original ACL footprint area appears to be beneficial for achieving ACL isometric reconstruction. However, researchers must be mindful of the aforementioned limitations when interpreting the results of this study.

## Data Availability

The original contributions presented in the study are included in the article/Supplementary Material, further inquiries can be directed to the corresponding authors.
